# Measurement of Healthy Adult Brain Temperature Using ^1^H Magnetic Resonance Spectroscopy Thermometry

**DOI:** 10.1007/s00062-024-01467-3

**Published:** 2024-10-30

**Authors:** Yahong Tan, Wenjia Liu, Yanhua Li, Nan Zhang, Mingxiao Wang, Shuo Sun, Lin Ma

**Affiliations:** 1 Medical School of Chinese PLA, Beijing, China; 2 Department of Radiology, First Medical Center, Chinese PLA General Hospital, 28 Fuxing Road, 100853 Beijing, China; 3 School of Medicine, Nankai University, Tianjin, China

**Keywords:** ^1^H Magnetic Resonance Spectroscopy, Temperature, Age, Brain

## Abstract

**Purpose:**

The purpose of this study is to measure the brain temperature (*T*_br_) by using ^1^H magnetic resonance spectroscopy (^1^H MRS) thermometry and investigate its age and gender differences in healthy adults. The brain temperature was further compared with the body temperature (*T*_bo_) to investigate the possible existence of brain-body temperature gradient (*∆T*).

**Methods:**

A total of 80 subjects were included in this study. ^1^H MRS data were collected on a 3.0T MR scanner using Point Resolved Selective Spectroscopy (PRESS) sequence. Voxels were positioned in the right frontal (RF) lobe and left frontal (LF) lobe, respectively. The temperature of each voxel was calculated by chemical shift difference (*∆δ*) between H_2_O and NAA which was obtained by LCModel software. The average temperature of bilateral frontal lobe voxels was defined as *T*_br_ for each subject. The average forehead temperature was acquired before MR scanning, defined as *T*_bo_, in this study. The difference between *T*_br_ and *T*_bo_, denoted as the brain-body temperature gradient (*∆T*), was calculated. Age and gender characteristics of *T*_br_, *∆T* and *T*_bo_ were analyzed.

**Results:**

T_br_ (38.51 ± 0.59℃) was higher than *T*_bo_ (36.47 ± 0.26℃) (*P* < 0.05). Negative correlations were observed between *T*_br_ and age (r = −0.49, *P* < 0.05) and between *∆T* and age (r = −0.44, *P* < 0.05), whereas no correlation existed between *T*_bo_ and age (r = −0.03, *P* = 0.79).

**Conclusion:**

Our observation demonstrated that the brain temperature, derived from ^1^H MRS thermometry, is significantly higher than the body temperature, indicating the existence of a brain-body temperature gradient, and the brain temperature gradually decreases with age.

## Introduction

The brain comprises only ~2% of adult human body mass, yet accounts for 25% of the body’s total glucose utilization (~ 5.6 mg glucose per 100 g of human brain tissue per minute) and 20% of oxygen consumption (~ 3–3.5 ml O_2_ per 100 g of human brain tissue per minute) in resting state [1, 2]. It is a high energy demand organ. Previous data have shown that the power consumption of a single central neuron is about 0.5–4.0 nW, which is about 300–2500 times more than the average somatic cells [3]. High-intensity heat production is one of the fundamental characteristics of brain metabolic activity since all energy used for brain metabolism is ultimately transformed into heat [4]. The heat remove of brain mainly depends on cerebral blood flow (CBF), and studies have proved that the temperature gradient in the brain (from higher to lower) is from brain parenchyma (heat source) to CBF (heat clearance) [5]. As one of the monitoring parameters for brain thermal homeostasis, brain temperature (*T*_br_) can indirectly reflect the metabolic intensity and CBF status. For instance, in patients with acute ischemic stroke, vascular obstruction disturbs the thermal homeostasis, and further lead to temperature elevation in brain [6]. Monitoring such changes in temperature holds crucial implications for guiding clinical intervention management for patients.

Despite brain temperature being a factor that reflects neural activity and affects various neurological functions, data on brain temperature in conscious individuals is lacking. In fact, direct methods for measuring *T*_br_, such as using implantable catheters [7], are challenging. Currently, these invasive methods are limited to apply in patients with traumatic brain injury [8]. The utilization of ^1^H MRS to measure *T*_br_ has gradually been developed and applied in human research. Compared with direct *T*_br_ measurement, ^1^H MRS has several advantages including non-invasive, free from ionizing radiation, and broader range population for application [9]. Moreover, ^1^H MRS thermometry exhibits higher accuracy, repeatability, and reliability with absolute value compared to other MR-based temperature measurement methods [10]. At present, there is no study describing age or gender characteristics of brain temperature in normal adult. In addition, body temperature (*T*_bo_) is generally divided into surface (e.g., forehead, axillary) temperature and core (e.g., esophagus, bladder, rectal) temperature in human. Since the forehead temperature is well consistent with the axillary temperature [11], the forehead temperature is defined as *T*_bo_ in this study.

The aim of the present study is to use ^1^H MRS to obtain *T*_br_ values in healthy subjects and evaluate the gender and age characteristics of brain temperature. We hypothesized that there exist temperature differences in age and gender, which can be revealed by ^1^H MRS thermometry. Additionally, the differences between *T*_br_ and *T*_bo_, ie brain-body temperature gradient (*∆T*), will be investigated.

## Materials and Methods

### Participants

Eighty healthy participants were recruited for this observational study with informed consent obtained from March 2022 to January 2024, including 40 females (age range: from 20 to 80 years) and 40 males (age range: from 20 to 80 years) without any previous history of brain disease or intracranial surgery. The exclusion criteria included pregnant individuals, those with a history of fever within three days prior to examination, and subjects who took antipyretic drugs (including steroids and non-steroidal anti-inflammatory drugs) or psychotropic drugs on the day of examination. Participants who showed any abnormalities in the brain on MRI were also excluded. The study was approved by the local medical ethical committee and conformed to standards set by the Declaration of Helsinki.

### Experimental Design

**Fig. 1:**
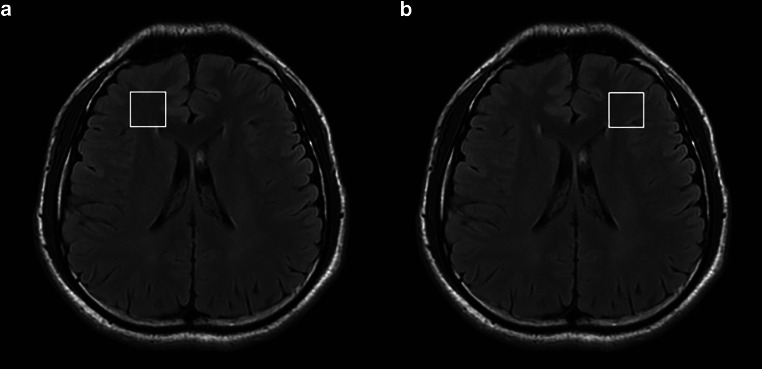
Illustration of voxel of interest using ^1^H MRS. **a** voxel location in right frontal lobe, **b** voxel location in left frontal lobe

^1^H MRS using Point Resolved Selective Spectroscopy (PRESS) sequence were performed, and two voxels were placed in the white matter of right frontal (RF) and left frontal (LF) lobes, respectively (Fig. 1). A total of 160 voxels were obtained for statistical analysis. The temperature for each voxel was derived from the ^1^H MRS data. The average temperature of both *T*_RF_ (RF lobe temperature) and *T*_LF_ (LF lobe temperature) was computed as *T*_br_. Forehead temperature, representing the body temperature (*T*_bo_), was measured using an electronic thermometer (FT85, BEURER, Beurer GrmBH, Germany), with three readings taken and averaged as *T*_bo_. Additionally, the weight and height of all participants were collected to calculate the Body Mass Index (BMI).

### MR Examination

All participants were required to remain still during the MR examination. A 3.0T MR scanner (Discovery 750, GE Healthcare, Milwaukee, WI, USA) and an 8‑channel head coil were used. MR scan included T2-FLAIR (fluid attenuated inversion recovery) and PRESS sequence. The parameters of T2-FLAIR were as follows: repetition time (TR) = 8500 ms, echo time (TE) = 162 ms, field of view (FOV) = 24 cm × 24 cm, bandwidth = 41.67 kHz, slice thickness = 6 mm, slice spacing = 1.5 mm, acquisition time = 2 min 17 sec. The parameters of PRESS were as follows: TR = 1500 ms, TE = 144 ms, voxel = 2 cm × 2 cm × 2 cm, numbers of excitations = 8, acquisition time = 3 min 48 sec.

### Temperature Calculation

The chemical shift difference (∆δ, ppm) between the water peak and the NAA peak in ^1^H MRS data was obtained using LCModel software (Linux Systems, Version 6.3-1R; http://lcmodel.ca/lcmodel.shtml). The ∆δ was processed in accordance with previous researches [12]. And the temperature of each voxel was calculated using the following equation [13, 14]: $\mathrm{T}=286.9-94\times \Delta \delta$, where T is the temperature (℃).

### Statistical Analysis

Statistical analysis was performed using SPSS software (version 17.0, SPSS, Chicago, IL, USA). OriginPro (Version 2021, OriginLab Corporation, Northampton, MA, USA) is used to create artwork. We estimated the sample size by using the G ∗ Power software version 3.1 and with an alpha level of 5% [15]. The temperature differences between brain and body, as well as between bilateral frontal lobes were analyzed using paired sample *t* test. Pearson correlation coefficients were used to analyze the correlation of *T*_br,_ *∆T*, and *T*_bo_ with age. The gender differences in *T*_br,_ *T*_bo_, and *∆T* were compared using independent sample *t* test. *P* < 0.05 was considered statistically significant. Quantitative data were presented as mean ± standard deviation (mean ± SD).

## Results

### General Data for All Participants

The age (female: 42.1 ± 15.0 years vs. male: 41.3 ± 11.9 years) and BMI (female: 23.65 ± 2.74 kg/m^2^ vs. male: 24.62 ± 1.94 kg/m^2^) showed no significant differences between females and males (*P* > 0.05).

### Temperature Measurement

**Fig. 2:**
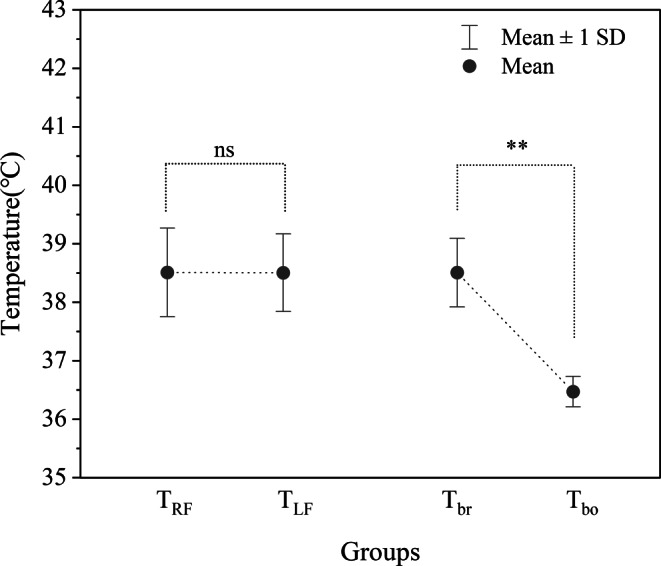
The differences between *T*_RF_ and *T*_LF_ and between *T*_br_ and *T*_bo_ in all subjects ns: *P* > 0.05, **: *P* < 0.05

There was no significant difference in temperature between RF lobe (38.51 ± 0.76℃) and LF lobe (38.50 ± 0.66℃) (*P* > 0.05) (Fig. 2). The average *T*_br_ (38.51 ± 0.59℃) was significantly higher than the average *T*_bo_ (36.47 ± 0.26℃) (*P* < 0.05), and there existed brain-body temperature gradient (*∆T*) (2.04 ± 0.64℃) (Fig. 2).

**Fig. 3:**
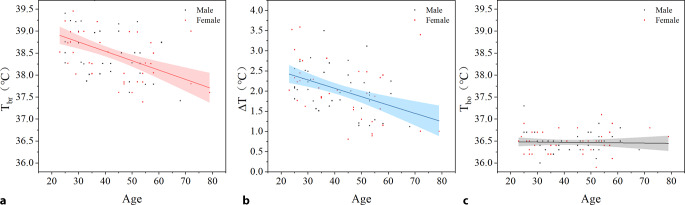
Correlation analysis of *T*_br_ (**a**), *∆T* (**b**), *T*_bo_ (**c**) with age in all subjects

The correlation of *T*_br,_ *∆T*, and *T*_bo_ with age were −0.49 (*P* < 0.05), −0.44 (*P* < 0.05), and −0.03 (*P* = 0.79), respectively (Fig. 3).

**Fig. 4:**
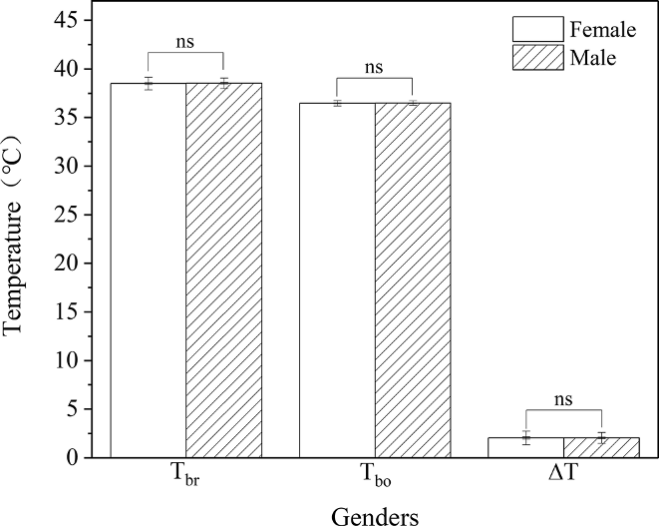
Gender differences of *T*_br_*,* *T*_bo_*,* *∆T* in all subjects ns: *P* > 0.05

*T*_br_ (female: 38.49 ± 0.64℃ vs male: 38.52 ± 0.54℃, *P* = 0.84), *T*_bo_ (female: 36.46 ± 0.28℃ vs male: 36.48 ± 0.24℃, *P* = 0.73), and *∆T* (female: 2.03 ± 0.71℃ vs male: 2.04 ± 0.56℃, *P* = 0.96) showed no significant differences between genders (Fig. 4).

## Discussion

### Brain Temperature Characteristics

In the present study, ^1^H MRS was used to measure the brain temperature of healthy subjects, and the findings revealed a negative correlation between brain temperature and age (r = −0.49, *P* < 0.05), indicating a decreasing trend in brain temperature with increasing age. Previous studies suggested that there is an interaction among brain temperature, energy metabolism, and CBF [1, 16]. Therefore, it is speculated that the decrease in brain temperature with age might be associated with age-related alterations in metabolic intensity and CBF. Existing data showed that brain heat production in healthy adults is approximately 0.66 J · g^−1^ · min^−1^ at rest state [16–18]. Generally, heat of organism dissipates through radiation, conduction, convection, and evaporation, but in central nervous system, the removal of heat relies on the washout of CBF [1, 17, 19]. Some researchers suggested that changes in blood flow affect *T*_br_ through changes in convective heat transfer in circulation [12, 18]. Aanerud et al. demonstrated a decrease in CBF and metabolic intensity with age, closely related to neurovascular coupling mechanisms [20]. This partially explains the observed decrease in *T*_br_ with age in our work. Despite the interactional relationship among *T*_br_, energy metabolism and CBF, the exact relationship between these factors cannot be determined due to the limited *T*_br_ data. Additionally, our results showed no statistical difference (*P* > 0.05) between *T*_RF_ (38.51 ± 0.76℃) and *T*_LF_ (38.50 ± 0.66℃) measured by ^1^H MRS, which is consistent with the previous research [21], implying that the brain itself maintains the stability of thermal distribution, which is a prerequisite for cerebral executive function.

### Brain-body Temperature Gradient

Before the application of brain temperature measurement, the oral, axillary, or forehead temperatures were used to predict brain temperature [22, 23]. However, there is evidence to support the hypothesis that brain temperature is higher than body temperature [24]. Our study once again demonstrated that brain temperature (38.51 ± 0.59℃) is significantly higher than body temperature (36.47 ± 0.26℃) (*P* < 0.05), proving the presence of brain-body temperature gradient (2.04 ± 0.64℃). In fact, Nybo et al. found that brain is established with a jugular-venous-to-arterial temperature difference of approximately 0.3℃ in a study involving human volunteers [25]. Similarly, direct measurement of the average temperature of cerebrospinal fluid (CSF) in the frontal horn of the lateral ventricle (4–5 cm below the brain surface) showed higher temperatures (0.3–0.9℃) than body temperature [26, 27]. Furthermore, a review analysis of 15 studies by Mcilvoy et al. revealed that, compared to body temperature, brain temperature tends to be higher with an average difference of 0.4℃–2.5℃ [28]. Although the magnitude of the brain-body temperature gradient varies, our results are consistent with the prior reports in healthy volunteers.

The understanding of brain-body temperature difference is crucial in clinical practice as timely detection of abnormal brain temperature could avoid further injury of neurons. For instance, Rumana et al. found that the average brain temperature in patients with traumatic brain injury (TBI) after 5 days was 38.9 ± 1.0℃, while the average rectal temperature was 37.8 ± 0.4℃. Notably, a body temperature of 37.8℃ is rarely considered fever in clinical practice, while temperatures exceeding 38.9℃ are often treated with cooling therapy [29]. Due to lack of data on normal brain temperature, it remains uncertain whether these brain-body temperature discrepancies could alter the management of patients with TBI. Nevertheless, clinicians should bear in mind the differences as they may affect the patient outcomes. Currently, brain temperature measurement using ^1^H MRS has become easily accessible. Monitoring of brain and core temperatures simultaneously could not only elucidate thermoregulatory characteristics but also aid clinicians in identifying and treating hyperthermia in patients with acute neural injuries. In order to minimize the influence in temperature gradients caused by distance factor, forehead temperature was selected as the body temperature in our study, which is the closest surface temperature monitoring point to the frontal lobe.

### Clinical and Experimental Implications

Firstly, as a promising brain temperature measurement method, non-invasive thermometry by ^1^H MRS can be applied in conscious individuals without MR contraindications. Secondly, the understanding of the relationship between normal brain temperature value and age/gender may help determine the causes of *T*_br_ changes and to eliminate research bias. Finally, *T*_br_ can be used to monitor the thermodynamic homeostasis of the brain, and investigation on *T*_br_ may help further elucidate the coupling mechanism between brain metabolism and CBF, which is essential for neuroscience research. Overall, brain temperature and brain-body temperature gradient need further exploration.

### Limitations

There are some potential limitations in this study. Firstly, the sample size of the study is relatively small, and validation with a larger sample population is still needed. Secondly, single voxel measurement was used in order to ensure the reliability and accuracy despite its lower spatial and temporal resolution compared to multi-voxel brain temperature measurement. Thirdly, we have not been able to obtain the real brain temperature of normal conscious individuals due to ethical issues, and currently the observation of real brain temperature is still a thorny issue. Finally, existing data suggest that the alterations of body temperature are related with menstrual cycle in females [30], our study did not include data on menstrual cycles in female participants and showed no gender differences in *T*_br_, and it remains unclear whether the menstrual cycle related temperature changes also exist in the brain.

## Conclusion

Our research found that brain temperature measured by ^1^H MRS is higher than body temperature, and there is a brain-body temperature gradient in healthy volunteers. Meanwhile, we observed that brain temperature gradually decreased with age. The normal brain temperature values acquired with ^1^H MRS thermometry will provide the basis for future clinical study for neurological diseases. Moreover, further research is still needed to have a better understanding of interaction among brain temperature, energy metabolism and CBF.
